# Author Correction: Twin-field quantum key distribution without optical frequency dissemination

**DOI:** 10.1038/s41467-023-40945-z

**Published:** 2023-08-29

**Authors:** Lai Zhou, Jinping Lin, Yumang Jing, Zhiliang Yuan

**Affiliations:** Beijing Academy of Quantum Information Sciences, Beijing, 100193 China

**Keywords:** Quantum optics, Fibre optics and optical communications, Quantum information

Correction to: *Nature Communications* 10.1038/s41467-023-36573-2, published online 18 February 2023

The original version of this Article contained an error in the first sentence of the 7th paragraph in the Results section, both in the HTML and in the PDF version, where the cited references incorrectly read:

“With the simplex channel stabilisation, we performed two sets of TF-QKD experiments using the SNS protocol [23,39,40].”

while the correct form should be:

“With the simplex channel stabilisation, we performed two sets of TF-QKD experiments using the SNS protocol [17,39,40].”

This has been corrected in both versions of the Article.

