# Supplementary material for: Twin-field quantum key distribution without optical frequency dissemination
Source: Nat Commun. 2023 Feb 18;14:928. doi: 10.1038/s41467-023-36573-2 (PMC9938887; doi:10.1038/s41467-023-36573-2)
Supplement: Supplementary file 1 — Supplementary Information [file 41467_2023_36573_MOESM1_ESM.pdf]

Supplementary Information
Twin-field quantum key distribution without optical frequency dissemination

Lai Zhou,[1] Jinping Lin,[1] Yumang Jing,[1] and Zhiliang Yuan[1,*]

[1]*Beijing Academy of Quantum Information Sciences, Beijing 100193, China*

Supplementary Note 1: Encoder

The purpose of the encoder is to turn the continuous-wave input into a 1 GHz, 300 ps pulse train with each pulse's intensity and phase set according to the requirements by a TF-QKD protocol. The encoder is capable of supporting all TF-QKD protocols, although just the SNS protocol was demonstrated in this work. Below, we describe the encoder with reference to Fig. 2**b**, Main Text.

The input light has its polarisation aligned to the slow axis of a series of intensity and phase modulators (3 IM's and 2 PM's), which are driven by a 25 GSa/s arbitrary waveform generator containing 2 waveform and 4 pattern output channels. IM_1 is modulated for carving out signal and decoy pulses and for extinguishing light transmission at vacuum time slots, IM_2 by a waveform signal for setting the signal/decoy intensity and further extinguishing the vacuum signals, and IM_3 by a second binary signal for adjusting the intensity contrast between the 'quantum reference' and the quantum signal. Together, three IM's prepare pulses of five different intensity levels: μ_{qr} (quantum reference), μ_Z (signal state), μ_2 (strong decoy), μ_1 (weak decoy) and μ_0 (vacuum). After intensity modulation, PM_1 and PM_2 encode the phase of each quantum signal pulse with one of 16 phase values, $\theta \in \{0, \pi/8, 2\pi/8...15\pi/8\}$ meeting TF-QKD's phase randomisation and qubit encoding requirement, while the quantum reference pulses are left unmodulated. The quantum signals are interleaved with the quantum reference in every 100 ns. Overall, the quantum signals occupy 50 % of the total time slots and have an effective clock rate of 500 MHz. In our experiments, we used a pattern length of 40000 bits, corresponding to a duration of 40 μs. Modulation patterns were carefully designed to produce correct sending and matching probabilities of different class of pulses as expected from the TF-QKD protocol.

* yuanzl@baqis.ac.cn

All optical elements in each encoder are installed in an enclosure for stability.

Supplementary Note 2: Active Feedbacks

To ensure stable operation, we implemented four feedback routines to correct the photon polarisation, the fast phase drift, the slow residual phase drift, and the laser frequency difference. These routines require each user to have two mutually coherent wavelengths of λ_c and λ_q for channel reference and quantum reference/signal.

As shown in Supplementary Fig. 1, each user's λ_q and λ_c signals of 100 GHz spacing are derived from the same laser via electro-optic frequency comb generation. They are separated by an optical filter with 55 dB isolation into two separate paths. The encoding path contains a fibre stretcher (FS), an electronically driven polarisation controller (EPC1) and a variable optical attenuator (VOA1) in addition to the modulation elements (not shown). The channel reference path is just a straight fibre. The two paths are multiplexed together by a 50 GHz Dense Wavelength Division Multiplexer (DWDM) into a second EPC (EPC2) and then sent to Charlie via the quantum link segment after being attenuated to the desired intensity by a second optical attenuator (VOA2). VOA1 sets the intensity contrast to a desired level between the two wavelengths, while VOA2 is to set the quantum signals to the correct fluxes as defined in Supplementary Table 4.

At each input, Charlie uses a polarisation beam splitter (PBS) to ensure all photons entering his 50/50 interfering beam splitter (BS) to have an identical polarisation and thus achieve maximum interference visibility. Polarisation rotation by the quantum link segment will affect the transmission through the PBS. The reflected signals by the PBS is routed to two single photon detectors (D_2 and D_3) via a polarisation beam combiner and a DWDM. D_2 (D_3) detects the λ_c (λ_q) photons and its count rate is minimised via controlling EPC2 (EPC1), thus maximising the transmissions. This feedback routine operates continuously at a rate of 5 - 10 Hz.

The interference outcome between Alice and Bob's channel references (λ_c) is detected by D_c and its count rate feeds to the FPGA PID controller which then computes a compensation voltage to drive Charlie's PM at a feedback rate of 200 kHz. This feedback locks Alice and Bob's channel reference signals to a differential phase of $\pi/2$, and corrects Alice and Bob's optical frequency difference and the fibre fluctuation of the entire quantum channel. The mutual coherence ensures the differential phase of the λ_q wavelength is mostly compensated

as well, with its residual amounting to about $|\lambda_q - \lambda_c|/\lambda_q$ of its original rate. This residual drift is then corrected for by Alice's FS with the feedback signal from D_1's count rate. This slow feedback operates at a rate of 50 – 100 Hz.

As described in Main Text (see Supplementary Fig. 3**c**), the FPGA PID controller allows instantaneous readout of the laser frequency difference even when the lasers are separated by hundreds of kilometers fibres. Once the frequency offset exceeds a certain value, *e.g.*, 300 Hz, Charlie transmits an instruction to Alice to offset her laser frequency accordingly. This feedback is applied less than daily thanks to the stability of the lasers.

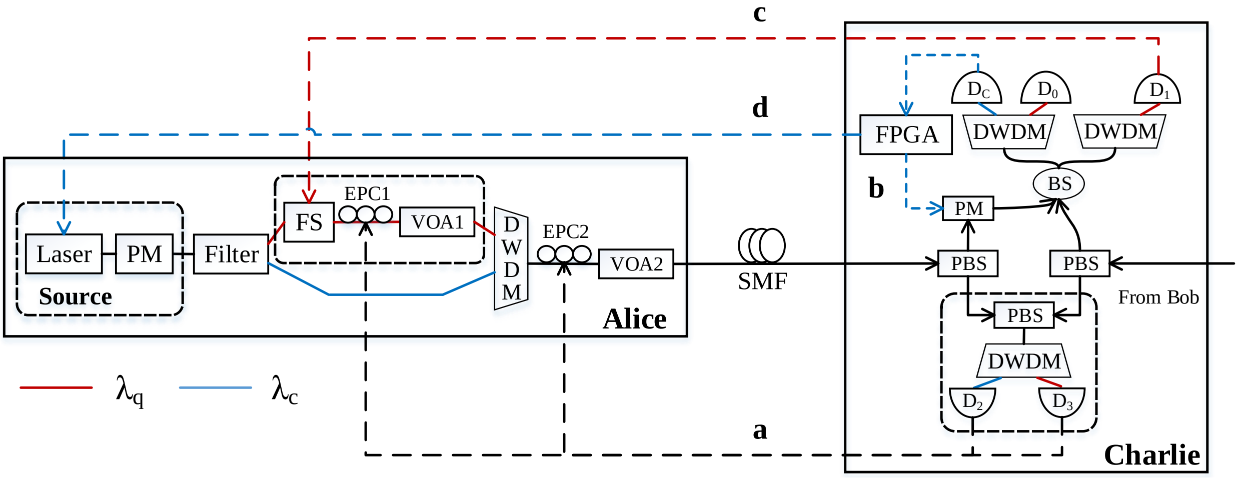

Supplementary Figure 1: Active feedbacks. Four feedback routines are implemented. **a** Polarisation precompensation using detectors D_2 and D_3 as error signals to adjust EPC2 and EPC1; Rate: 5 – 10 Hz. **b** Fast phase compensation for the channel reference λ_c using detector D_c as error signal to drive Charlie's phase modulator (PM); Rate: 200 kHz; **c** Active phase compensation for the quantum wavelength λ_q using detector D_1's count rate as error signal to adjust Alice's fibre stretcher (FS); Rate: 50 – 100 Hz; **d** Active correction of the laser frequency difference. Rate: daily. Routine a applies on both Alice and Bob, b locally at Charlie, c and d apply just on Alice. BS: beam splitter; DWDM: dense wavelength division multiplexer; EPC: electronically driven polarisation controller; PBS: polarisation beam splitter/combiner; PM: phase modulator; SMF: single mode fibre; VOA: variable optical attenuator.

Supplementary Note 3: System loss and noise characterisation

In our setup, the quantum channel is made of ultra-low-loss fibre spools (Corning G654.C ULL) of mainly 25.2 km and 50.4 km each in length. They were spliced into sets of different

lengths to allow varying fibre lengths in experiments. Including loss from fibre connectors, the quantum channel was characterised to have an average loss coefficient of 0.168 dB km^{-1}. Supplementary Table 1 summarises the lengths and their corresponding losses for the fibre configurations used in our experiments.

Inside Charlie's module, there is a chain of fibre components from either user's input port to the single photon detectors. These components and their losses are summarised in Supplementary Table 2. Charlie's transmission loss at Alice's side is 4.5 dB, which is 2 dB higher than Bob's side because of the extra phase modulator. This 2 dB asymmetry was compensated either by a 2 dB attenuator in the symmetric 403.73 km experiment or by a 10 km fibre spool for all other experiments.

We present the performance of superconducting nanowire single photon detectors (SNSPD's: D_0 and D_1) in Supplementary Table 3. These SNSPDs are polarisation-sensitive, so a manual polarisation controller is placed in front of each SNSPD for maximising the efficiency. Their noise counts include both detector dark counts (2 Hz) and scattered noise (2 Hz), measured for the longest fibre channel of 615.59 km when the count rate of D_c was kept at 13 MHz. This level of noise corresponds to a channel isolation of >68 dB of λ_q from λ_c at Charlie.

Supplementary Table 1: Lengths and corresponding losses for the fibre links we used in the two sets of experiments.

	Total		Alice		Bob	
	length (km)	loss (dB)	length (km)	loss (dB)	length (km)	loss (dB)
Symmetric	403.73	67.89	201.87	33.97	201.86	33.92
	518.16	87.02	254.38	42.57	263.78	44.45
	615.59	103.27	302.80	50.55	312.79	52.72
Asymmetric	455.65	76.68	201.87	34.16	253.78	42.52
	407.23	68.55	153.45	26.03	253.78	42.52

Supplementary Note 4: Visibility characterisation

In this section, we describe how we measured the various interference visibilities reported in the Main Text.

Supplementary Table 2: Charlie's components loss.

	Alice	Bob
Polarisation beam splitter	0.7	0.7
Phase modulator	2.0	n/a
50/50 beam splitter	0.3	0.3
DWDM filter	1.2	1.2
Polarisation controller	0.3	0.3
Total loss (dB)	4.5	2.5

Supplementary Table 3: Performance of Charlie's detectors D_0 and D_1. The detector noise was characterised with 615 km fibre channel and a channel reference count of 13 MHz.

Detector	Efficiency	Noise Count Rate
D_0	60 %	4 Hz
D_1	65 %	4 Hz

To measure the interference visibility over a free-drifting long quantum channel, we use an optical power meter with a sampling rate of 500 kHz. Each measurement has a duration of 10 s and produces a corresponding data array of 5×10^6 samples. We compute a visibility (V) for each segment of 2×10^4 samples (40 ms) from the average ($\bar{I}_{max}$) of the 5 highest values and the average ($\bar{I}_{min}$) of the 5 lowest values,

$$V = \frac{\bar{I}_{max} - \bar{I}_{min}}{\bar{I}_{max}+\bar{I}_{min}}. \tag{1}$$

We then calculate the average visibility from the entire 10 s data and its standard deviation.

Supplementary Fig. 2 shows the measured interference fringes for the channel reference over the free-drifting quantum channel of 615.6 km. When Alice and Bob shared a common laser (**a**), the measured visibility is just 99 % and this imperfect visibility is caused mainly by the 10 km asymmetry in the quantum link. The visibility drops to 97.8 % when Alice and Bob use truly independent lasers with nominally 0 Hz offset (**b**), illustrating the performance penalty by the loss of mutual frequency locking. Increasing the offset to 2 kHz (**c**, **d**), the measured visibility further decreases to ∼97.0 %. However, this further decrease is attributed mainly to the limitation of the power meter sampling rate, because the absolute drift rate is higher in the presence of a frequency offset, as shown in Fig. 3**b** of the Main Text. Note

that Supplementary Figs. 2**c** and **d** can also have destructive interference occasionally as low as those lowest in **b** when the net drift rate happens to be slow.

Once the quantum channel is stabilised by the FPGA PID controller, the residual phase drift of the quantum signal becomes much slower. In this case, we use a power meter at a slower sampling rate of 200 Hz. We record a 50 s data containing 10^4 samples. From every 10 s of data (2000 samples), we compute a visibility value from averaging 10 highest and 10 lowest values, and then compute the average visibility and its standard deviation. Supplementary Fig. 3 reports the measurement result for the 615.6 km quantum channel with two independent lasers. The average visibility is 96.8 %.

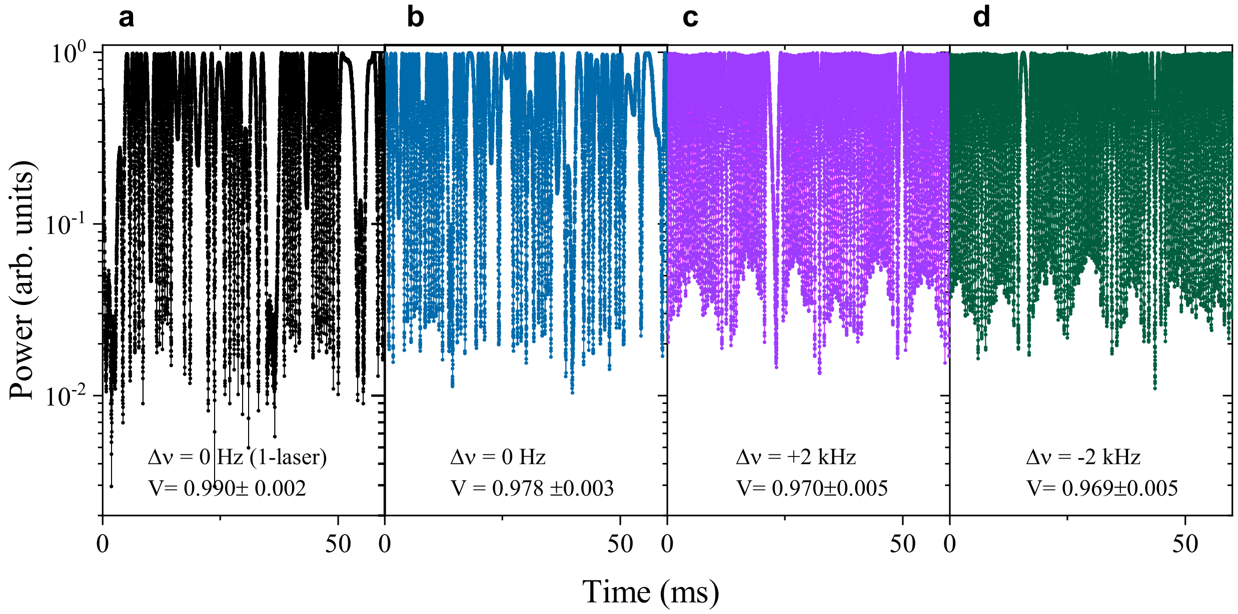

Supplementary Figure 2: Free-drifting interference fringes. All data were measured with the 615.6 km (302.8 km + 312.8 km) that drifts freely and a power meter at a sampling rate of 500 kHz. **a** Alice and Bob shared a common laser; **b** Independent lasers with nominally 0-Hz frequency offset; **c** As b but with +2 kHz frequency offset; **d** As b but with -2 kHz offset.

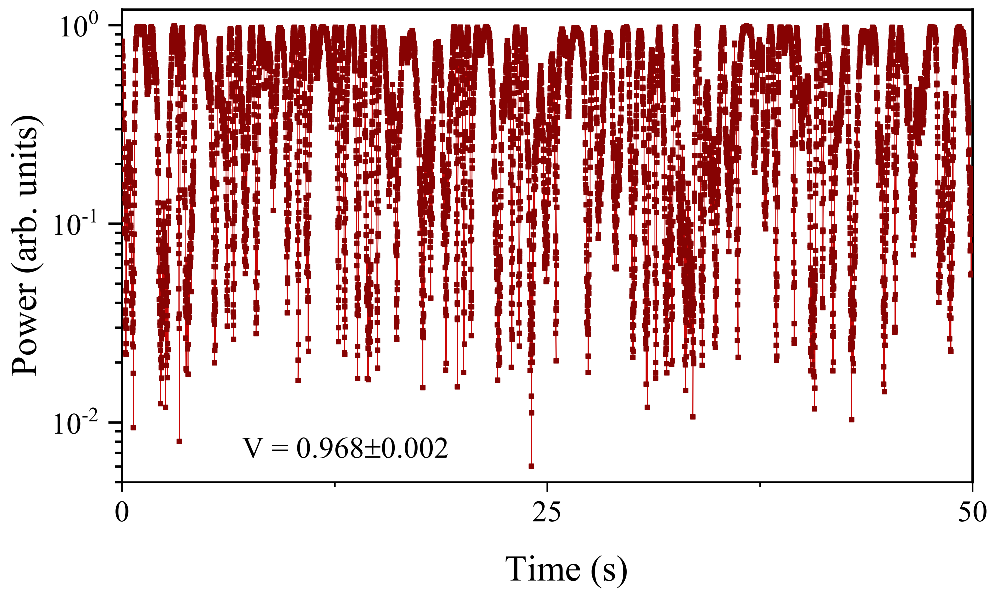

Supplementary Figure 3: Interference fringes of the quantum wavelength. The 615.6 km (302.8 km + 312.8 km) was stabilised by the FPGA PID controller and the data were recorded by a power meter at 200 Hz. Alice and Bob used independent lasers with nominally 0 Hz detuning. An average interference visibility of $\mathrm{V} = 0.968 \pm 0.002$ was obtained.

Supplementary Note 5: Detailed experimental parameters and results

In Supplementary Table 4, we give the detailed experimental encoding parameters used for symmetrical and asymmetrical setups. The respective mean photon numbers for the quantum reference are 0.53, 3.02 and 13.1 photon/pulse for fibre lengths of 403.73, 518.16 and 615.59 km. Supplementary Table 5 and 6 summarize the experimental results and quantities relative to the key rate calculation using AOPP for symmetric and asymmetric cases, respectively. The total number of signal pulses sent is given by N_{tot}. The number of valid detection events reported by Charlie is denoted as "Detected $\mathrm{AB_{ab}}$", where "A" ("B") is X or Z indicating the basis Alice (Bob) chooses; "a" ("b") is 0, 1, 2 or 3 indicating the intensity Alice (Bob) chooses is μ_{A0} (μ_{B0}), μ_{A1} (μ_{B1}), μ_{A2} (μ_{B2}), or μ_{Az} (μ_{Bz}), respectively. "QBER ($\mathrm{X_{11}}$)" and "QBER ($\mathrm{X_{22}}$)" represent the error rates in X bases for the decoy states with respective intensity "11" and "22". The rest of the notations is explained in the main text.

Supplementary Table 4: Encoding parameters used in the two sets of experiments. In symmetric experiments, Alice and Bob share an identical set of parameters. μ_Z, μ_2, μ_1 and μ_0 are the values of mean photon number per pulse for signal, strong decoy, weak decoy and vacuum pulses. P_Z is the probability of a pulse to be encoded in the coding (Z) basis. The parameter ϵ is the probability of a signal pulse to be actually sent when Z basis is chosen. $P_X = 1 - P_Z$ is the selection probability of the checking (X) basis, while p_{μ_2}, p_{μ_1} and p_{μ_0} are the selection probabilities for preparing μ_2, μ_1 and μ_0 decoy states in the X-encoding basis.

Parameter	Symmetric 403.7km/518.2km/615.6km	Asymmetric 407.2km/455.7km	
	Alice/Bob	Alice	Bob
μ_Z	0.493	0.493	0.493
μ_2	0.493	0.114	0.493
μ_1	0.105	0.057	0.246
μ_0	0.0002	0.0002	0.0002
P_Z	0.735	0.735	0.735
P_X	0.265	0.265	0.265
ϵ	0.269	0.136	0.405
p_{μ_2}	0.216	0.216	0.216
p_{μ_1}	0.706	0.706	0.706
p_{μ_0}	0.078	0.078	0.078

Supplementary Table 5: Finite-size symmetric SNS with AOPP: experimental results at various quantum link fibre lengths.

Total length (km)	403.73	518.16	615.59
Alice - Charlie (km)	201.87	254.38	302.80
Bob - Charlie (km)	201.86	263.78	312.79
N_{tot}	2.025×10^{12}	2.475×10^{12}	1.4175×10^{13}
Number of phase slices	16	16	16
Detected XX_{20}	93574	15168	13360
Detected XX_{02}	102956	11292	18185
Detected XX_{10}	60774	11428	11201
Detected XX_{01}	63251	12807	12698
Detected XX_{00}	38	14	85
Detected XZ_{00}	814	358	1464
Detected XZ_{10}	1656903	296696	310666
Detected XZ_{20}	2336341	418785	402898
Detected ZX_{00}	788	389	1624
Detected ZX_{01}	1650969	293079	323169
Detected ZX_{02}	2333340	418695	419565
Detected ZZ_{03}	8020164	1420140	1492968
Detected ZZ_{30}	8390554	1440863	1400694
Detected ZZ_{33}	6121571	1082752	1050251
Detected ZZ_{00}	21033	9178	40051
QBER (X_{11})	5.17%	4.81%	4.75%
QBER (X_{22})	4.77%	5.02%	5.12%
QBER (E_z before AOPP)	27.24%	27.62%	27.37%
QBER (E_z after AOPP)	0.19%	0.48%	1.97%
n_1 (Before AOPP)	9.565×10^6	1.665×10^6	1.744×10^6
n_1 (After AOPP)	1.616×10^6	2.763×10^5	2.984×10^5
e_1^{ph} (Before AOPP)	7.09%	7.57%	6.29%
e_1^{ph} (After AOPP)	13.36%	14.49%	12.24%
SKR (bit/s)	146.70	14.38	0.32
SKR (bit/signal)	2.934×10^{-7}	2.875×10^{-8}	6.366×10^{-10}
SKC_0 (bit/signal)	2.380×10^{-7}	2.845×10^{-9}	6.565×10^{-11}
Ratio SKR over SKC_0	1.23	10.11	9.70

Supplementary Table 6: Finite-size asymmetric SNS with AOPP: experimental results at various quantum link fibre lengths.

Total length (km)	407.23	455.65
Alice - Charlie (km)	153.45	201.87
Bob - Charlie (km)	253.78	253.78
N_{tot}	2.25×10^{12}	2.2×10^{12}
Number of phase slices	16	16
Detected XX_{20}	20887	27708
Detected XX_{02}	19018	20497
Detected XX_{10}	37937	36554
Detected XX_{01}	36669	33340
Detected XX_{00}	20	27
Detected XZ_{00}	369	453
Detected XZ_{10}	785947	804659
Detected XZ_{20}	474298	492710
Detected ZX_{00}	610	558
Detected ZX_{01}	1118620	1192466
Detected ZX_{02}	669280	699462
Detected ZZ_{03}	3499529	3672181
Detected ZZ_{30}	3611502	3679227
Detected ZZ_{33}	2956649	2999841
Detected ZZ_{00}	10955	12133
QBER (X_{11})	5.31%	5.07%
QBER (X_{22})	5.10%	5.19%
QBER (E_z before AOPP)	29.44%	29.06%
QBER (E_z after AOPP)	0.26%	0.27%
n_1 (Before AOPP)	4.251×10^6	4.354×10^6
n_1 (After AOPP)	6.963×10^5	7.084×10^5
e_1^{ph} (Before AOPP)	8.21%	7.74%
e_1^{ph} (After AOPP)	15.43%	14.60%
SKR (bit/s)	46.31	50.75
SKR (bit/signal)	9.261×10^{-8}	1.015×10^{-7}
SKC_0 (bit/signal)	2.078×10^{-7}	3.193×10^{-8}
Ratio SKR over SKC_0	0.45	3.18
